# Charge density waves and degenerate modes in exfoliated monolayer 2H-TaS_2_

**DOI:** 10.1107/S2052252520011021

**Published:** 2020-08-25

**Authors:** Duan Zhang, Yecun Wu, Yu-Hsin Su, Ming-Chien Hsu, Cormac Ó Coileáin, Jiung Cho, Miri Choi, Byong Sun Chun, Yao Guo, Ching-Ray Chang, Han-Chun Wu

**Affiliations:** a Elementary Educational College, Beijing Key Laboratory for Nano-Photonics and Nano-Structure, Capital Normal University, Beijing 100048, People’s Republic of China; b School of Physics, Beijing Institute of Technology, Beijing 100081, People’s Republic of China; c Department of Physics, National Taiwan University, Taipei 106, Taiwan; d Department of Physics, National Sun Yat-sen University, Kaohsiung 80424, Taiwan; e Western Seoul Center, Korean Basic Science Institute, Seoul 03579, Republic of Korea; f Chuncheon Center, Korean Basic Science Institute, Chuncheon 24341, Republic of Korea; g Division of Industrial Metrology, Korea Research Institute of Standards and Science, Daejeon 3050340, Republic of Korea

**Keywords:** 2H-TaS_2_, charge density waves, transition metal dichalcogenides, periodic lattice distortion, degenerate modes

## Abstract

Charge density waves spontaneously breaking lattice symmetry through periodic lattice distortion, and electron–electron and electron–phonon inter­actions, can lead to a new type of electronic band structure. Bulk 2H-TaS_2_ is an archetypal transition metal dichalcogenide supporting charge density waves with a phase transition at 75 K. Here, it is shown that charge density waves can exist in exfoliated monolayer 2H-TaS_2_ and the transition temperature can reach 140 K, which is much higher than that in the bulk. The degenerate breathing and wiggle modes of 2H-TaS_2_ originating from the periodic lattice distortion are probed by optical methods. The results open an avenue to investigating charge density wave phases in two-dimensional transition metal dichalcogenides and will be helpful for understanding and designing devices based on charge density waves.

## Abstract 2

Using temperature-dependent Raman scattering, it is demonstrated for the first time that charge density waves can exist in exfoliated monolayer 2H-TaS_2_ with a phase transition temperature much higher than that in the bulk.

## 1. Introduction   

Structural charge density waves (CDWs), transitions from a normal to a distorted phase, have been extensively studied for many decades (Grüner, 1988 ▸). Such transitions can be characterized by the competing influences of the energy cost associated with distortion of the crystal structure and liberation due to the opening of an electronic energy gap. CDW transitions are known to occur in a wide variety of materials, a prominent example being layered transition metal dichalcogenides (TMDs), which have received much attention due to their novel optical and electronic properties. The properties of TMDs often show a strong dimensional dependence, including a CDW phase transition (Xi *et al.*, 2015 ▸; Yu *et al.*, 2015 ▸). It has been suggested that it is possible to tune the CDW transition temperature of thin TMDs through dimensionality (Xi *et al.*, 2015 ▸), electrostatic gating (Yu *et al.*, 2015 ▸) or strain engineering (Gao *et al.*, 2018 ▸). This would enable the quantum phase transitions to be controlled in a manner that is compatible with current semiconductor technology.

Tantalum di­sulfide, TaS_2_, is an archetypal TMD material which exhibits CDWs (Yu *et al.*, 2015 ▸). TaS_2_ consists of layers stacked by weak van der Waals bonding, with each covalently bound layer typically consisting of a sheet of hexagonally arranged Ta atoms sandwiched between two S layers. 1T-TaS_2_ shows a (13)^1/2^ × (13)^1/2^ CDW below 540 K. Upon cooling from 540 K, 1T-TaS_2_ undergoes several CDW transitions, and may change from incommensurate to nearly commensurate at 350 K and then to commensurate at 180 K (Zwick *et al.*, 1998 ▸). Bulk 2H-TaS_2_ forms a 3 × 3 commensurate CDW at low temperature (Thompson *et al.*, 1972 ▸) and undergoes an incommensurate in-plane CDW transition at about 78 K (Scholz *et al.*, 1982 ▸; Yoshida *et al.*, 2014 ▸) and a superconducting transition at about 0.8 K (Nagata *et al.*, 1992 ▸). Previous studies of 1T-TaS_2_ have shown the importance of dimensionality for the CDW phase transitions (Yu *et al.*, 2015 ▸; Yoshida *et al.*, 2014 ▸). It was found that as the thickness was reduced, the transition from the nearly commensurate to the commensurate CDW phase shifts towards lower temperatures during cool-down and suddenly vanishes for a critical thickness. Recently, Albertini *et al.* (2016 ▸) further reported that the commensurate CDW phase was the ground state even for monolayer 1T-TaS_2_.

Although the thickness-dependent CDW in 1T-TaS_2_ has been studied intensively, very few studies have focused on the CDW structure in thin exfoliated 2H-TaS_2_ (a few layers or even a monolayer). In particular, Albertini *et al.* (2016 ▸) indicated that 1T-TaS_2_ retains its inversion symmetry regardless of the number of layers, but the symmetry of 2H-TaS_2_ is dependent on its number of layers. Here it is worth noting that for an even-layer 2H-TaS_2_ structure, there is an inversion symmetry, whereas for an odd-layer 2H-TaS_2_ structure, including the monolayer 2H-TaS_2_ structure in the present study, there is a non-inversion symmetry. Moreover, CDWs in TaS_2_ spontaneously breaking the lattice symmetry through periodic lattice distortion, or electron–electron and electron–phonon interactions, may lead to some new types of electronic structure. Thus, optical methods such as Raman spectroscopy would provide a non-destructive and easy way of probing the CDWs in thin exfoliated 2H-TaS_2_.

In this work, based on density functional simulations, we demonstrate that CDWs can exist in exfoliated monolayer 2H-TaS_2_ and the transition temperature is much higher than that in the bulk. A new peak appears at 155 cm^−1^ below the transition temperature, which corresponds to the breathing mode and wiggle mode of the CDW of 2H-TaS_2_, suggesting that the CDW transition and periodic lattice distortion can be probed and determined by optical methods, such as temperature-dependent Raman scattering.

## 2. Methods   

### 2.1. Sample preparation and characterizations   

TaS_2_ nanosheets of different thicknesses were mechanically exfoliated from bulk 2H-TaS_2_ purchased from HQ Graphene. Optical colour contrast and Seiko SPI3800N atomic force microscopy (AFM) measurements were combined to identify the thicknesses of the nanosheets. Temperature-dependent Raman spectra were taken using a Bruker Senterra confocal spectrometer with an excitation wavelength of 532 nm. High-resolution transmission electron microscopy (HRTEM) was performed using a JEOL transmission electron microscope.

### 2.2. Phonon dispersion calculations   

Phonon dispersion calculations for bulk and monolayer 2H-TaS_2_ were carried out using a supercell approach (Parlinski *et al.*, 1997 ▸) with the *PHONOPY* code (Togo *et al.*, 2008 ▸). Before executing the *PHONOPY* package (Togo & Tanaka, 2015 ▸), the fully relaxed structures were obtained from the *VASP* relaxation procedure (Sholl & Steckel, 2009 ▸). To optimize the bulk and monolayer 2H-TaS_2_ by employing *VASP*, the energy cut-off of the plane wave expansion was set to 500 eV, the *k* points adopted from the Monkhorst–Pack method were set to be 16 × 16 × 4 for the bulk structure and 16 × 16 × 1 for the monolayer structure, and the energy and atomic force convergence criteria for self-consistency were set to be 10^−9^ eV and 10^−6^ eV Å^−1^, respectively. The van der Waals force interaction was taken into account. For the phonon dispersion calculations, the size of the supercell was chosen as 4 × 4 × 4 for bulk and 4 × 4 × 1 for monolayer 2H-TaS_2_, respectively. In addition, a 2 × 2 × 1 supercell extension for calculating the phonon dispersion of a 3 × 3 × 1 unit-cell structure was selected where the CDW phase of monolayer 2H-TaS_2_ existed.

## 3. Results and discussion   

**Figure 1:**
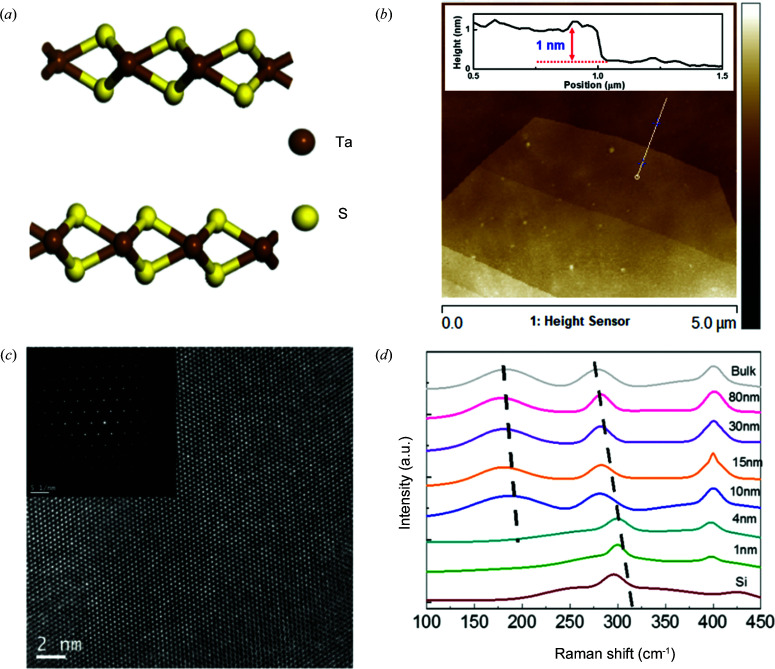
TaS_2_ characterizations. (*a*) A schematic drawing of the atomic structure of TaS_2_. (*b*) An AFM image of mechanically exfoliated TaS_2_ flakes with a thickness of about 1 nm. (*c*) An HRTEM image of TaS_2_ flakes. Inset: SAED of TaS_2_. (*d*) Raman spectra of mechanically exfoliated TaS_2_ flakes of various thicknesses. The excitation wavelength is 532 nm.

In the present study, 2H-TaS_2_ nanosheets of different thicknesses (from 1 nm to over 100 nm) were exfoliated from a commercially grown 2H-TaS_2_ single crystal and then transferred onto SiO_2_/Si substrates using Scotch tape. Fig. 1 ▸(*a*) shows the atomic structure of 2H-TaS_2_, where the Ta atoms are in a trigonal prismatic coordination with the S atoms. AFM, transmission electron microscopy (TEM) and Raman spectroscopy were used to investigate the thickness and quality of the exfoliated 2H-TaS_2_ nanosheets. As shown in Fig. 1 ▸(*b*), the smooth AFM image of the exfoliated 2H-TaS_2_ nanosheet indicates the layered structure. The cross-sectional height reveals that the thickness of the exfoliated TaS_2_ film is about 1 nm. The high-resolution TEM image [Fig. 1 ▸(*c*)] and corresponding selected-area electron diffraction (SAED) [inset of Fig. 1 ▸(*c*)] of TaS_2_ demonstrate the single-crystal hexagonal structure and high quality of the exfoliated sample. The unit-cell distance *d* = 2.85 Å indicates that the exposed surface is the (100) plane of 2H-TaS_2_.

Fig. 1 ▸(*d*) displays the Raman spectra of 2H-TaS_2_ for various thicknesses, excited by a 532 nm laser line in an ambient environment. The Raman spectra of thick 2H-TaS_2_ are consistent with previous reports (Sugai *et al.*, 1981 ▸; Hangyo *et al.*, 1983 ▸), and the Raman data for the ultrathin sample are shown here for the first time to the best of our knowledge. *A*_1*g*_ (∼400 cm^−1^ for bulk TaS_2_) and 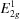 (∼280 cm^−1^ for bulk TaS_2_) modes are observed in both ultrathin and bulk TaS_2_. The other two modes (*E*_1*g*_, 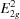) could not be detected, either because of the selection rules for our scattering geometry (*E*_1*g*_) or because of the limited rejection of the Rayleigh scattered radiation (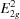). Remarkably, a strong band peaking at ∼180 cm^−1^ is observed for thick samples due to second-order scattering. With increasing number of layers, the interlayer van der Waals force in 2H-TaS_2_ suppresses the out-of-plane vibration, so both the second-order scattering and 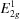 mode are stiffened (blue shift). However, the red shift of the *A*_1*g*_ mode indicates that long-range Coulombic interlayer interactions may dominate the variation of the Raman mode, which is consistent with many other 2D materials (Zhang *et al.*, 2016 ▸). Noticeably, the Raman data for the thin sample (<4 nm) show two significant differences with respect to the thicker samples, where the second-order scattering peak degenerates and the 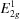 mode shows a dramatic red shift.

**Figure 2:**
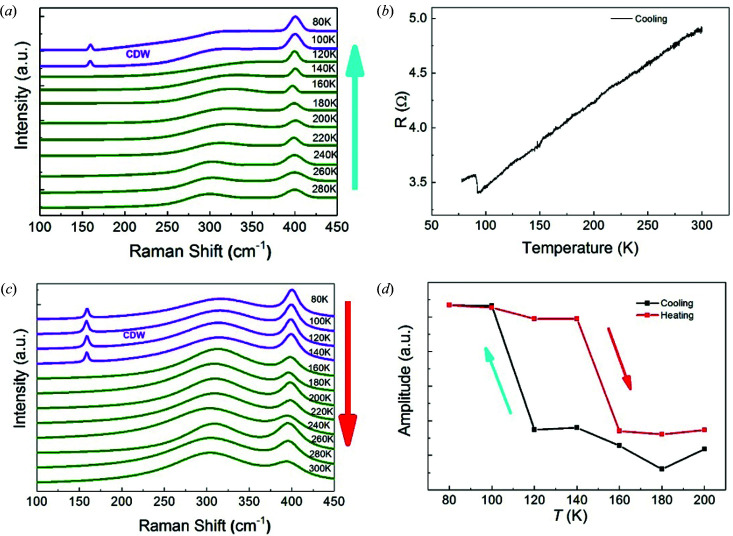
Probing CDWs in monolayer TaS_2_. (*a*) Raman spectra for monolayer TaS_2_ acquired during the cooling cycle. (*b*) Resistivity measurements, showing a temperature-induced phase transition. (*c*) Raman spectra for the same monolayer TaS_2_ acquired during the heating cycle. (*d*) The temperature dependence of the Raman intensity for the Raman mode at 400 cm^−1^.

Fig. 2 ▸(*a*) shows the temperature evolution of the Raman spectra of the 2H-TaS_2_ monolayer measured at the same position during a cooling cycle. With decreasing temperature, the 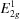 mode shows a red shift. Interestingly, apart from the peaks of the *A*_1*g*_ and 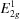 modes, a new peak appears at ∼155 cm^−1^ when the temperature is below 100 K. Bulk 2H-TaS_2_ undergoes a phase transition at 75 K and the distorted CDW phase is formed below the transition temperature *T*_c_ (Tidman *et al.*, 1974 ▸). The appearance of the new peak may be due to the formation of the CDW in monolayer 2H-TaS_2_ at low temperature. To confirm this, the temperature-dependent electrical resistance curve was plotted for monolayer 2H-TaS_2_ during cooling, as shown in Fig. 2 ▸(*b*). A sudden jump in resistance is observed at 93 K, indicating that the CDW phase transition occurs even in monolayer 2H-TaS_2_ and the transition temperature is higher than that in the bulk. The increased *T*_c_ may be due to the reduced dimensionality, which enhances electron–phonon coupling and has been observed in other 2D CDWs (Xi *et al.*, 2015 ▸; Chen *et al.*, 2016 ▸; Goli *et al.*, 2012 ▸). Fig. 2 ▸(*c*) further shows the Raman spectra of the same monolayer 2H-TaS_2_ during the heating cycle. Remarkably, the new peak can be clearly observed even at 140 K. Interestingly, a huge hysteresis of almost 60 K was measured, which was also observed for 1T-TaS_2_ (Sun *et al.*, 2018 ▸; Scruby *et al.*, 1975 ▸; Yoshida *et al.*, 2014 ▸; Tsen *et al.*, 2015 ▸; Wang *et al.*, 2018 ▸). Fig. 2 ▸(*d*) summarizes the intensity of the *A*_1*g*_ mode as a function of temperature. It is found that the intensity of the *A*_1*g*_ mode also shows a similar thermal hysteresis effect. We also measured 2H-TaS_2_ flakes of other thicknesses (Fig. S1 in the supporting information) and found a similar effect. This hysteresis could be related to a first-order transition which shows a visible discontinuous change in unit-cell parameters, as reported in previous studies (Sun *et al.*, 2018 ▸; Scruby *et al.*, 1975 ▸; Yoshida *et al.*, 2014 ▸; Tsen *et al.*, 2015 ▸; Wang *et al.*, 2018 ▸). Furthermore, the occurrence of this new peak (∼155 cm^−1^) in both monolayer and thick 2H-TaS_2_ with CDWs indicates that the vibration mode is Raman active regardless of the number of layers, which is also confirmed by our theoretical analysis of the vibration mode presented below.

Additionally, we can gain some insight into the commensurability of CDWs from temperature-dependent Raman spectroscopy. It is generally recognized that the commensurability of CDWs can change with temperature (Fu *et al.*, 2020 ▸), but in the present work we find that the intensity of the CDW-related Raman peak (∼155 cm^−1^) is stable with changing temperature, implying that the CDWs observed in this experiment are probably completely commensurable. We therefore stress that the present work is the first to indicate that CDWs can exist in monolayer 2H-TaS_2_.

To provide further confirmation that the peak (∼155 cm^−1^) appearing at low temperature is the result of the formation of CDWs, we calculated the phonon dispersion for lattice dynamics of bulk and monolayer 2H-TaS_2_ with and without the CDW phase based on density functional theory (Kresse & Hafner, 1993 ▸; Kresse & Joubert, 1999 ▸; Blöchl, 1994 ▸) and the *PHONOPY* code (Parlinski *et al.*, 1997 ▸; Togo *et al.*, 2008 ▸; Togo & Tanaka, 2015 ▸). Details of the simulations can be found in the *Methods* [Sec sec2] section.

**Figure 3:**
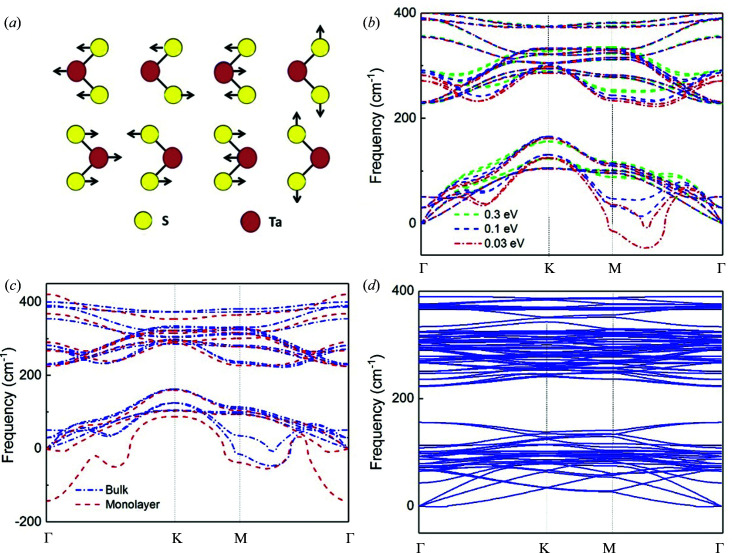
Phonon dispersion of TaS_2_. (*a*) A schematic diagram of (from left to right) the 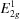, *E*_1*g*_, 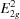 and *A*_1*g*_ Raman active modes of bulk 2H-TaS_2_. (*b*) Phonon dispersion of bulk 2H-TaS_2_ as a function of the electronic smearing parameter σ. (*c*) Phonon dispersion of bulk and monolayer 2H-TaS_2_ with a smearing parameter σ = 0.03 eV. (*d*) Phonon dispersion of monolayer 2H-TaS_2_ with a 3 × 3 × 1 unit cell.

Two molecular units of TaS_2_ compose the unit cell of bulk 2H-TaS_2_. The irreducible representations of the vibrational modes for bulk 2H-TaS_2_ in the *D*_6*h*_ space group are: *A*_1*g*_ + 2*B*_2*g*_ + *E*_1*g*_ + 2*E*_2*g*_ + 2*A*_2*u*_ + *B*_1*u*_ + 2*E*_1*u*_ + *E*_2*u*_, where 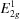, *E*_1*g*_, 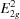 and *A*_1*g*_ are Raman active modes (Zhang *et al.*, 2016 ▸), as shown in Fig. 3 ▸(*a*). Fig. 3 ▸(*b*) shows the phonon dispersion of bulk 2H-TaS_2_. Among them, three bands belong to the acoustic branch and fifteen bands belong to the optical branch. Interestingly, with a smearing parameter σ = 0.03 eV, a segment of the acoustic branch indicates a negative phonon frequency, which is less than approximately 50 cm^−1^ along the M–Γ direction. A similar phenomenon also occurs in monolayer 2H-TaS_2_, as shown in Fig. 3 ▸(*c*), where the maximum negative frequency approaches 150 cm^−1^ along the M–Γ–K direction.

In further calculations, the smearing parameter physically represents the electronic temperature and can qualitatively affect the phonon properties of the material by considering temperature effects. Hence, the dependence of phonon bands on the smearing parameter indicates that the large negative phonon frequencies along the acoustic branches will eventually be overshadowed and become wholly positive as smearing increases with temperature.

The emergence of negative phonon frequencies along the M–Γ–K direction in both bulk and monolayer 2H-TaS_2_ provides a clue to obtain the phonon dispersion of monolayer 2H-TaS_2_ in the CDW phase. Physically, negative phonon frequencies represent an unstable mechanical structure. To obtain the stable structure of monolayer 2H-TaS_2_ and remove negative phonon frequencies, the unit cell should be extended along the Γ–M direction. Significantly, such an extension of the unit cell along the Γ–M direction basically behaves like the experimentally observed CDW phase in bulk 2H-TaS_2_, where the CDW phase is close to a supercell of 3 × 3 × 1 of the unit-cell structure (Sugai, 1985 ▸; Harper *et al.*, 1977 ▸). Fig. 3 ▸(*d*) shows the phonon dispersion of monolayer 2H-TaS_2_ with a 3 × 3 × 1 unit cell. The negative phonon frequencies have completely vanished. This firmly demonstrates that a 3 × 3 × 1 unit cell structure is mechanically stable. More importantly, two distinct phonon frequencies emerge at about 155 cm^−1^ at the Γ point, which are not observed for bulk 2H-TaS_2_. From the above simulation results, one can confirm that the CDW phase of monolayer 2H-TaS_2_ truly and stably exists in a 3 × 3 × 1 unit-cell structure. Moreover, the two CDW-induced frequencies at ∼155 cm^−1^ from our numerical simulation coincide very well with the experimental results of the Raman spectra, as shown in Fig. 2 ▸.

To summarize, for 2H-TaS_2_ with a 1 × 1 × 1 unit cell, one cannot obtain the CDW peak at ∼155 cm^−1^, while for 2H-TaS_2_ with a 3 × 3 × 1 unit cell, two CDW modes appear at frequencies of 155.6691 cm^−1^ and 155.6718 cm^−1^ from *ab initio* calculations, which are very close to the experimental observations near 155 cm^−1^. The two CDW modes near 155 cm^−1^ are shown in animations (see supporting information for details). One is a ‘breathing’ mode with the Ta atoms coming closer to and moving further away from the central S atoms, and the other is a ‘wiggle’ mode with the Ta atoms wiggling back and forth around the S atoms (Amelinckx, 1971 ▸). The breathing and wiggle CDW modes can be viewed as degenerate modes.

**Figure 4:**
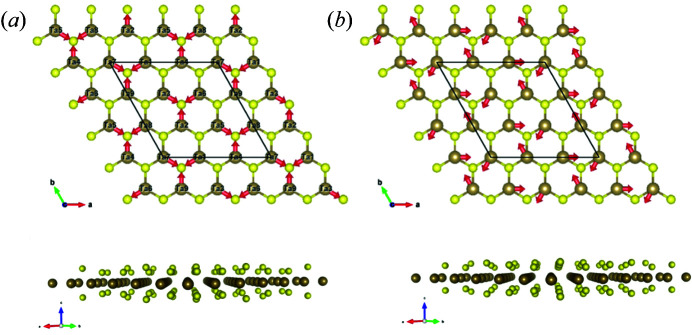
The degenerate modes of CDWs in TaS_2_. Top and side views of the atom displacements are shown for the degenerate (*a*) breathing CDW mode and (*b*) wiggle CDW mode. Yellow denotes the sulfur atoms and brown denotes the tantalum atoms.

**Figure 5:**
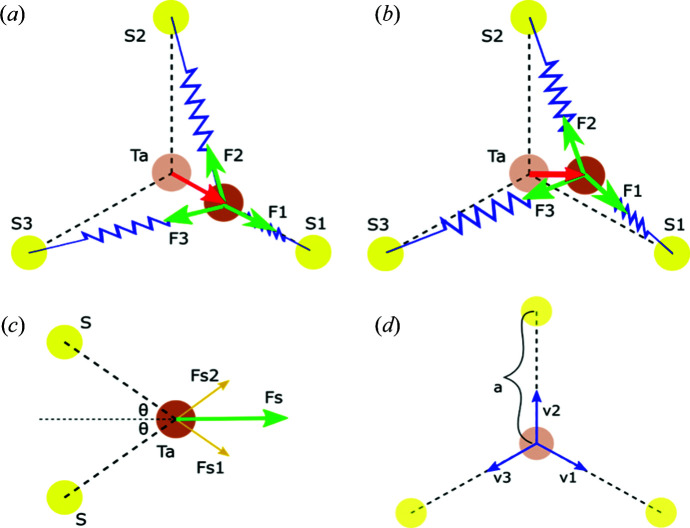
The origin of the degenerate modes of CDWs in TaS_2_. Top views of the force contributions from the nearby atoms for (*a*) breathing mode and (*b*) wiggle mode. (*c*) A side view of the force contributions from two nearby S atoms on top of each other. (*d*) The definition of the in-plane vectors along the undistorted lattice bonds.

It is worth discussing the origin of the degeneracy of the oscillation frequency for these two modes. To show the vibrational patterns clearly, it is necessary to consider a cell size three times larger in both the *a* and *b* directions for the CDW 3 × 3 × 1 unit cell. Fig. 4 ▸ shows top-down views of the structure, with the directions of the atomic displacements indicated for both the breathing and wiggle modes. The arrows denote the directions along which the atoms oscillate back and forth. Realistically, both Ta and S atoms oscillate, but the displacement is comparatively small for S so we only need to consider the movement of the Ta atoms. Although the Ta atoms apparently oscillate in very different manners for the breathing and wiggle modes, they are indeed the same at the larger scale if we consider the collective motion of individual nearest-atom triangular sub-units defined by three S atoms around one Ta atom. This equivalence can be more clearly recognized if we shift the origin of the wiggle mode by −*b*/3. Therefore, to analyze the origin of the degenerate frequencies, we only need to focus on the triangular cells, as shown in Fig. 5 ▸. The oscillations are regulated by the force exerted by the restorative potentials and can effectively be treated as a system or lattice of Ta atoms connected by several springs. As the lowest-order approximation, we need to consider only the nearest S atoms affecting the spring forces from the Ta—S bonds, and all the spring constants are the same since the S atoms are equidistant. As seen, there are six S atoms around each Ta atom. Since for 2H-TaS_2_ there is a honeycomb structure from the top view, three S atoms are situated above the other three S atoms, and thus the movements of the Ta atoms for both modes are in-plane and the S atoms can be further simplified by treating them as three in-plane atoms. If the spring constant between the actual S atoms and the Ta atom is *K*, the combined force of the two vertically aligned S atoms is 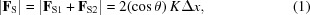
[see Fig. 5 ▸(*c*)], where |**F**_S1_| = |**F**_S2_| = *K*Δ*x* and Δ*x* is the atomic displacement of Ta from its equilibrium point. The contribution from two vertically aligned S atoms is equivalent to an in-plane atom with an effective spring constant *k* = 2(cos θ) *K*.

For the breathing mode shown in Fig. 5 ▸(*a*), the Ta atoms oscillate along the line connecting a Ta and an S atom. The net force exerted on the displaced Ta atom is the sum of the spring force from the three neighbouring atoms, which is **F** = **F**_1_ + **F**_2_ + **F**_3_, where 1, 2 and 3 label the three simplified S atoms and their respective directions. The Ta atom is shifted towards S1 by Δ*x*, and thus **F**_1_ = −*k*Δ*x* **v**_1_, where **v**_1_ is the unit vector towards atom S1 [Fig. 5 ▸(*d*)]. Considering the lowest-order approximation, the spring constant is assumed to be isotropic. Therefore, the effective displacement with respect to atom S2 is 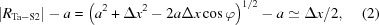
and its direction is along **v**_2_ since the displacement Δ*x* is very small. Similarly, the effective displacement from atom S3 is Δ*x*/2 along **v**_3_. Therefore, their spring forces are 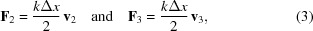
respectively. The total spring force on the Ta atom is therefore 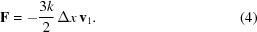



Assuming the Ta atom has a mass *m*, the oscillation frequency for the breathing mode is obtained as 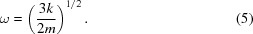
Displacement along **v**_2_ and **v**_3_ would be equivalent. For the wiggle mode, Ta atom displacement is not directed towards the S atoms but rather towards the next adjacent Ta atom. Performing the same geometric analysis in this case, the displacement is along the *x* direction and the forces due to the three simplified S atoms are 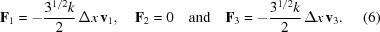
Therefore, the resultant force is
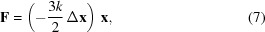
where **x** is the unit vector along the unit-cell vector **a**_1_. Therefore, the oscillation frequency for the wiggle mode is still 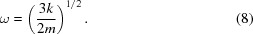



Finally, it should be indicated that at the lowest order, both the breathing and wiggle modes are degenerate, displaying the same oscillation frequency. It is also noted that we have assumed that the three Ta—S bonds have the same bond energy, thus conferring the degeneracy of the breathing and wiggle modes. However, because internal effects (*e.g.* defects) or external effects (*e.g.* applied stress) can introduce anisotropy into the CDW modes, the degeneracy could be lifted, the three Ta atoms could vibrate incoherently and several nearby peaks could be observed around 155.6 cm^−1^.

## 4. Conclusions   

Nondestructive Raman spectra evidence for the existence of a charge density wave (CDW) in monolayer 2H-TaS_2_ has been obtained. The CDW shows a much higher transition temperature than in the bulk structure and further results in additional vibrational modes, indicating strong interactions with light. Since several light-tunable devices have been proposed recently based on the CDW phase transition of 1T-TaS_2_ (Zhu *et al.*, 2018 ▸; Vaskivskyi *et al.*, 2015 ▸), the present study could provide a thorough understanding and further design principles for such devices based on the CDW of 2H-TaS_2_.

## Supplementary Material

Additional figures. DOI: 10.1107/S2052252520011021/gq5013sup1.pdf

Animation of breathing mode. DOI: 10.1107/S2052252520011021/gq5013sup2.mp4

Animation of wiggle mode. DOI: 10.1107/S2052252520011021/gq5013sup3.mp4
