# Supplementary material for: Charge density waves and degenerate modes in exfoliated monolayer 2H-TaS2
Source: IUCrJ. 2020 Aug 25;7(Pt 5):913–9. doi: 10.1107/S2052252520011021 (PMC7467171; doi:10.1107/S2052252520011021)
Supplement: Supplementary file 1 [file m-07-00913-sup1.pdf]

IUCrJ

Volume 7 (2020)

Supporting information for article:

Charge density waves and degenerate modes in exfoliated monolayer 2H-TaS_2

Duan Zhang, Yecun Wu, Yu-Hsin Su, Ming-Chien Hsu, Cormac Ó Coileáin, Jiung Cho, Miri Choi, Byong Sun Chun, Yao Guo, Ching-Ray Chang and Han-Chun Wu

S1. Supplementary Figures

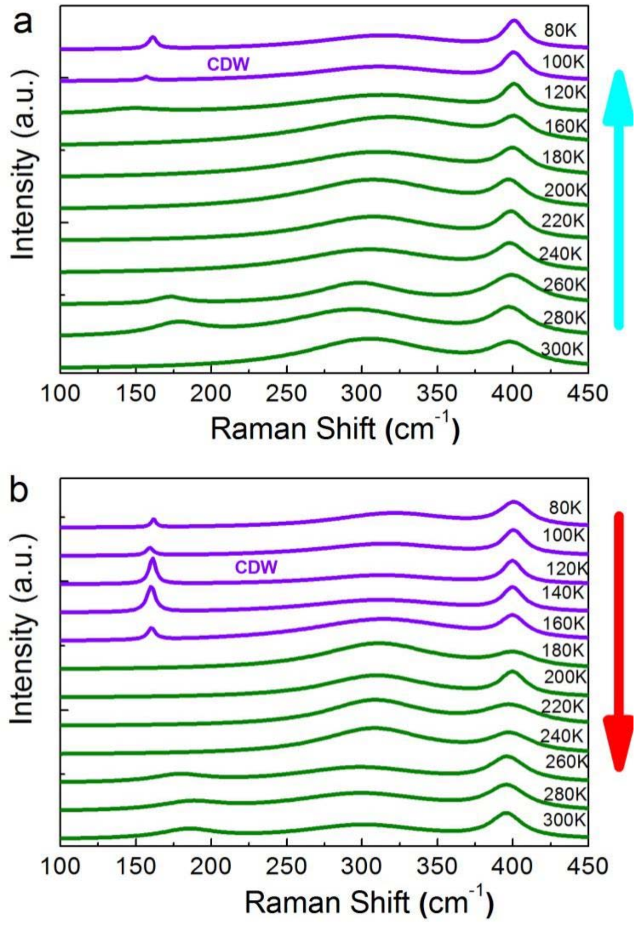

Figure S1 Probing CDW in 10 nm thick TaS_2. Raman spectra for 10 nm thick TaS_2 acquired during (a) cooling and (b) warming cycles.

By employing the *Phonopy* package, we can spatially visualize the atomic vibrations at a particular k point with a specific phonon band. Therefore, the figures for two degenerate CDW modes in 3×3×1 unit cell structure of monolayer 2H-TaS_2 are displayed. The corresponding animations are available as separate files.

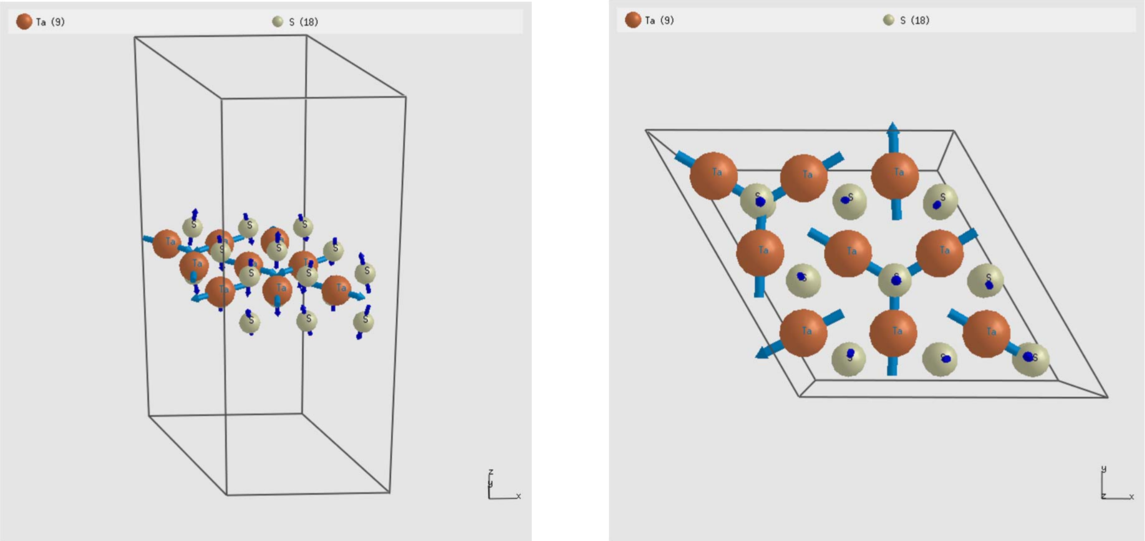

Figure S2 The degenerate breathing motion (Breathing mode) of CDW mode with frequency f=155.6691 cm^{-1}. The right figure shows the top view of the breathing mode.

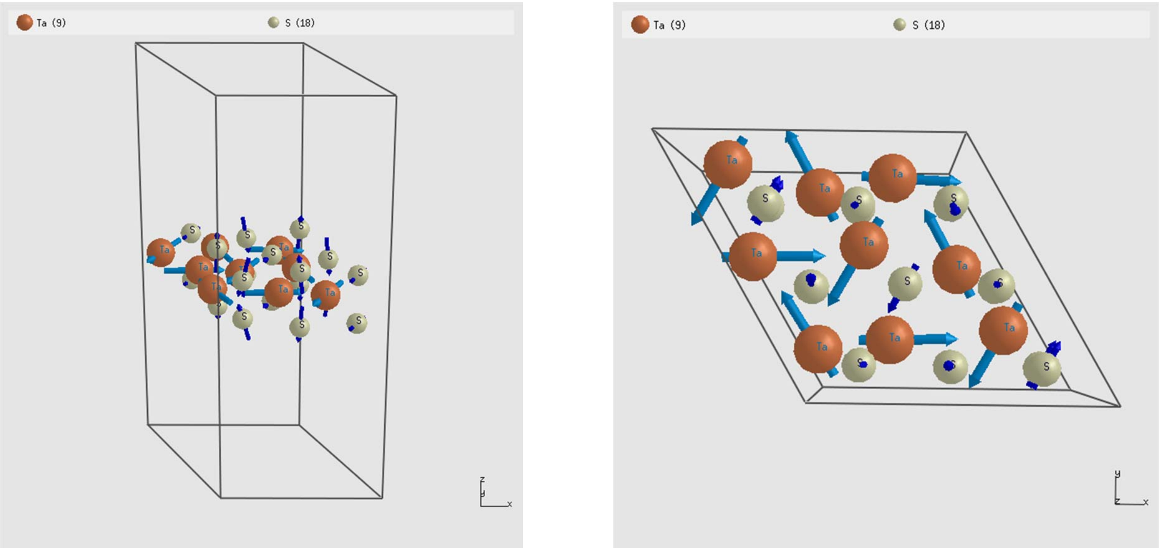

Figure S3 The degenerate wiggling motion (Wiggle mode) of CDW mode with frequency f=155.6718 cm^{-1}. The right figure shows the top view of the Wiggle mode.
